# Editorial: The insights of multi-omics into the microenvironment after tumor metastasis: a paradigm shift in molecular targeting modeling and immunotherapy for advanced cancer patients

**DOI:** 10.3389/fonc.2025.1749178

**Published:** 2025-12-16

**Authors:** Xuejian Liu, Qi Wang, Yang Ma, Xinhua Xiao, Xue Zhao, Hailin Tang, Divya Gopinath, Chao Wang

**Affiliations:** 1 Department of Radiology, Xinjiang 474 Hospital, Urumqi, China; 2 Department of Gastroenterology, Affiliated Hospital of Jiangsu University, Jiangsu University, Zhenjiang, China; 3 Department of General Surgery, Pancreatic Disease Center, Ruijin Hospital, Shanghai Jiao Tong University School of Medicine, Shanghai, China; 4 Shanghai Key Laboratory of Pancreatic Neoplasms Translational Research, Research Institute of Pancreatic Diseases, Shanghai Jiao Tong University School of Medicine, Shanghai, China; 5 State Key Laboratory of Systems Medicine for Cancer, Shanghai, China; 6 Institute of Translational Medicine, Shanghai Jiao Tong University, Shanghai, China; 7 Department of Hematology and Oncology, Women and Children's Medical Center, Guangzhou Medical University, Guangzhou, China; 8 Department of General Practice, Changshou Community Health Service Center of Putuo District, Shanghai, China; 9 State Key Laboratory of Oncology in South China, Guangdong Provincial Clinical Research Center for Cancer, Sun Yat-sen University Cancer Center, Guangzhou, China; 10 Basic Medical and Dental Sciences Department, College of Dentistry, Ajman University, Ajman, United Arab Emirates; 11 Centre of Medical and Bio-allied Health Sciences Research, Ajman University, Ajman, United Arab Emirates; 12 Department of Oncology, Ruijin Hospital, Shanghai Jiao Tong University School of Medicine, Shanghai, China

**Keywords:** immunotherapy, metastasis, microenvironment, multi-omics, tumor

Understanding the complex interplay within the tumor microenvironment (TME) following metastasis, using multi-omics technologies, is essential for revolutionizing treatments for advanced cancer. The post-metastatic TME is an intricate system of diverse cellular and molecular components (1). Integrating genomic, epigenomic, proteomic, and metabolomic data provides a panoramic view of the mechanisms driving tumor metastasis and therapy resistance. This holistic perspective facilitates the identification of molecular signatures and metabolic changes that are critical for developing precision medicine strategies (2–4). Such comprehensive analyses can yield innovative molecular targeting models and immunotherapeutic approaches, with the ultimate aim of transforming the therapeutic landscape for patients with advanced cancer (5).

This Research Topic aims to explore the transformative potential of multi-omics for understanding and treating advanced cancer. By dissecting the molecular intricacies and immune cell interactions within the post-metastatic tumor microenvironment (TME), this Research Topic seeks to advance therapeutic strategies that target both tumor cells and their surrounding milieu (6). These strategies are designed to enhance the efficacy of immunotherapy, improve patient survival rates, and increase quality of life. Driving this shift toward customized treatments represents an emerging frontier in oncology (7). To gather further insights into the intersection of immunotherapy, multi-omics, and the TME, we welcome contributions on key themes, including high-quality reviews on immunotherapy applications, investigations into the dynamic changes of the post-metastatic TME, multi-omics-guided optimization of immunotherapy, and biomarker validation for personalized medicine. This Research Topic, titled “Multi-Omics Insights into the Post-Metastatic Microenvironment: A Paradigm Shift for Molecular Targeting and Immunotherapy,” was curated by five guest editors and comprises 20 articles (5 reviews and 15 original research studies). Together, these works provide a novel, comprehensive perspective on the multi-omics-based dissection of the post-metastatic TME and propose potential precision strategies to overcome the challenges of advanced cancer.

A series of studies in this Research Topic examine the molecular regulatory mechanisms of the metastatic tumor microenvironment (TME) and the development of targeted immunotherapies to address the core challenges of immune escape and therapy resistance in advanced cancers. Gao et al. reviewed multi-omics findings on the cellular composition, signaling pathways, immune landscape, and metabolic rewiring within the metastatic TME, providing a comprehensive framework for using integrative omics data to guide personalized immunotherapeutic and targeted strategies. In colorectal cancer (CRC), Wang et al. reframed antibody-drug conjugates (ADCs) as immuno-oncology agents, demonstrating that payloads which induce immunogenic cell death or pyroptosis can convert the “cold” microsatellite-stable (MSS) CRC TME into an immunologically “hot” state, thereby synergizing with checkpoint inhibitors to overcome therapeutic resistance. Complementing this, Yang et al. identified the CCL28-STAT3-PLAC8 axis in CRC, showing that CCL28 activates STAT3 signaling to upregulate PLAC8, which in turn drives epithelial-mesenchymal transition (EMT) and metastasis; PLAC8 was also identified as an independent prognostic factor in CRC patients. Beyond CRC, Zhao et al. summarized the immunomodulatory role of γ-aminobutyric acid (GABA) in the TME, detailing how it regulates tumor-associated macrophages, CD8+ T cells, and dendritic cells to promote immune escape and immunotherapy resistance across multiple cancer types. In head and neck squamous cell carcinoma (HNSCC), Zhao et al. proposed a “Trinity” immune evasion network comprising metabolic reprogramming, stromal cell dysfunction, and epigenetic remodeling. Based on single-cell sequencing data, they further advanced a “lineage plasticity-driven immune adaptation” paradigm, offering novel insights into the immune escape mechanisms of HNSCC. Collectively, these studies delineate the complex regulatory networks of the metastatic TME and propose actionable immunotherapeutic strategies, laying a solid mechanistic foundation for transforming the treatment of advanced cancers.

Another group of studies focuses on constructing prognostic models and validating key biomarkers for advanced cancers by leveraging multi-omics data and machine learning to enhance prognostic precision and identify potential therapeutic targets. In breast cancer, Feng et al. identified 753 differentially expressed sodium-overload-related genes (DESORGs), built a prognostic ridge regression model centered on IFNG, and validated NR1H3 as a protective biomarker. For hepatocellular carcinoma (HCC), Gao et al. developed a stemness- and hypoxia-related prognostic index (SHRPI) using random forest and Cox regression analyses. They identified G6PD as a key regulator of hypoxia-driven cancer stemness, demonstrating that it interacts with HIF-1α to form a positive feedback loop under hypoxic conditions; they also proposed BI2536 as a promising therapeutic for high-SHRPI patients. In colorectal cancer (CRC), Zheng et al. established a lymph node-independent metastasis gene (LIMG) signature using data-independent acquisition mass spectrometry (DIA-MS) and machine learning, pinpointing ITGA11 as a key driver of early metastasis. For cervical cancer, Qin et al. integrated transcriptomic, mutational, and clinical data from The Cancer Genome Atlas (TCGA) and Gene Expression Omnibus (GEO) to construct a prognostic model. This model included high-risk biomarkers (EZH2, PCNA, BIRC5) and protective factors (CD34, ROBO4, CXCL12), and predicted greater sensitivity to Afuresertib and Venetoclax in high-risk patients. Additionally, Wang et al. combined a retrospective study with Mendelian randomization analysis, revealing that sarcopenia is associated with a poor prognosis in advanced CRC patients treated with fruquintinib and identifying the SYK gene as a key mediator of this treatment-associated sarcopenia. Collectively, these studies establish reliable prognostic tools and pinpoint critical molecular targets, thereby laying a foundation for personalized risk stratification and targeted therapy in advanced cancers.

A significant portion of the included studies is dedicated to developing non-invasive diagnostic and predictive tools and to optimizing clinical regimens, addressing the need to minimize invasive procedures and improve therapeutic efficacy. In lung cancer, Liu et al. developed a radiomics framework that integrated longitudinal CT tumor growth kinetics with deep learning to derive an Immune Evasion Score (IES). This score non-invasively predicts PD-L1 expression (AUC = 0.85), CD8+ T-cell exclusion, and immunotherapy response with high accuracy. For breast ductal carcinoma *in situ* (DCIS), Sha et al. created an interpretable Gradient Boosting Machine (GBM) model that combines mammographic deep learning features with clinicopathological data, achieving an AUC of 0.918 for predicting recurrence more than five years after lumpectomy; the mammographic signature, Ki-67 index, and histological grade were key predictors. In a related study, Pan et al. used a DCE-MRI radiomics model based on logistic regression to predict STAT3 expression in breast cancer. They found that high radiomics scores correlated with elevated STAT3 expression, longer overall survival, and an enhanced immune response, offering a non-invasive method for assessing STAT3-related tumor microenvironment features. For differentiated thyroid carcinoma (DTC), Wen et al. evaluated serological indicators and the pan-immune-inflammation value (PIV), demonstrating that a panel including TSH, FT4, Tg, and PIV achieved an AUC of 0.860 for diagnosis, underscoring PIV’s potential as a novel immune-inflammatory biomarker. In localized prostate cancer, Xu et al. used propensity score matching to compare high-dose and standard-dose stereotactic body radiotherapy (SBRT), finding that dose escalation did not significantly improve overall, cancer-specific, or biochemical progression-free survival, despite providing better local control. In CRC patients receiving neoadjuvant radiotherapy, Zhang et al. investigated radiation-induced intestinal barrier damage, observing impaired mucosal structure, elevated inflammatory factors in tissue and serum, and altered salivary metabolites. These findings inform the optimal timing for post-radiotherapy surgery. Another study by Zhang et al., combining retrospective analysis with meta-analysis, established that a high baseline spleen volume and an increase in volume during immune checkpoint inhibitor (ICI) therapy are independent predictors of poor overall and progression-free survival in HCC patients, providing a simple, imaging-based prognostic indicator for ICI therapy. Collectively, these non-invasive tools and optimized strategies help bridge the translational gap between basic research and clinical application, enhancing the accuracy of decision-making and the safety of interventions for advanced cancers.

A unique study in this Research Topic investigates precision therapy for uveal melanoma (UM) by integrating single-cell omics with materials science to address the disease’s high metastatic potential and poor prognosis. Fu et al. employed single-cell RNA sequencing (scRNA-seq), single-cell ATAC-seq (scATAC-seq), and spatial transcriptomics to characterize UM’s tumor heterogeneity, immunosuppressive tumor microenvironment (TME), and key molecular drivers—including novel macrophage subsets, senescent endothelial cells, and non-canonical immune checkpoints. Building on these omics insights, the authors integrated advances from materials science and biomedical engineering, demonstrating how engineered nanocarriers, biodegradable implants, and advanced gene therapy vectors could facilitate targeted drug delivery and genetic modulation tailored to the eye’s unique anatomy and immune privilege. The study further cataloged validated molecular targets for UM and proposed an interdisciplinary framework combining targeted therapies, immunomodulation, minimally invasive devices, and engineered delivery systems. This integrative approach transcends conventional oncology research boundaries, presenting a new paradigm for developing precision therapies for cancers with significant metastatic potential and unique anatomical constraints, while providing a blueprint for bench-to-bedside translation in UM.

The studies in this Research Topic collectively demonstrate the transformative power of multi-omics technologies in deciphering the metastatic tumor microenvironment (TME). By identifying novel molecular pathways and prognostic signatures and by developing non-invasive diagnostic tools and precision therapies, these works significantly advance our understanding of metastatic mechanisms and their clinical translation. Key advancements include the integration of multi-omics data with machine learning for robust modeling, the discovery of context-specific therapeutic targets, and cross-disciplinary synergy with materials science for targeted drug delivery. Notably, several studies highlight the critical role of the immunosuppressive TME in therapy resistance, underscoring the necessity for combination strategies that target both malignant cells and their microenvironment.

Future research should prioritize the large-scale validation of candidate biomarkers and targets, the development of real-time monitoring tools for the metastatic tumor microenvironment (TME), and clinical trials for multi-targeted combination therapies (8, 9). Furthermore, addressing the inter- and intratumoral heterogeneity of metastatic lesions is essential for advancing personalized medicine (10). We anticipate that the insights from this Research Topic will catalyze innovation in molecular targeting and immunotherapy, ultimately improving outcomes for patients with advanced cancer.
